# Challenges of microRNA‐based biomarkers in clinical application for cardiovascular diseases

**DOI:** 10.1002/ctm2.585

**Published:** 2022-02-15

**Authors:** David de Gonzalo‐Calvo, Jennifer Pérez‐Boza, Joao Curado, Yvan Devaux, EU‐CardioRNA COST Action CA17129

**Affiliations:** ^1^ Translational Research in Respiratory Medicine University Hospital Arnau de Vilanova and Santa Maria Lleida Spain; ^2^ CIBER of Respiratory Diseases (CIBERES) Institute of Health Carlos III Madrid Spain; ^3^ Flomics Biotech Barcelona Spain; ^4^ Cardiovascular Research Unit Luxembourg Institute of Health Luxembourg Luxembourg

Emerging omics technologies are providing innovative tools for medical‐decision making. A search of the current scientific literature clearly indicates that among transcriptomic biomarkers, microRNAs (miRNAs) are the most promising. A wide array of studies suggests that the circulating miRNA signature reflects the physiological or pathological status of a subject. Accordingly, the analysis of miRNA patterns may not only lead to novel approaches for diagnosis, prognostication, and selection of appropriate therapies, but also may help to understand the molecular pathways that mediate the pathology and regulate the interindividual variability to medical treatments.^1^

From a technical point of view, miRNAs may have the optimal biochemical properties to become easily accessible indicators. These small transcripts are highly stable, have a long half‐life in biological samples, their analysis does not require any special handling and can be applied to samples currently available. miRNAs can be quantified with relatively low cost, high sensitivity, and high specificity through standard techniques already employed in clinical laboratories such as quantitative PCR.

Several limitations have slowed down the application of circulating miRNAs in clinical practice. Here, we address the main challenges for the translation of miRNA‐based biomarkers to guide the management of the patient, with a special focus on the cardiovascular arena.

## 1 HIGH HETEROGENEITY OF METHODOLOGIES AND LACK OF CONTROLS

The lack of consensus regarding methodologies used for miRNA quantification is one of the main limiting factors in the application of these transcripts.

Several studies have proposed the use of circulating miRNAs as biological markers of the acute coronary syndrome,^2^ coronary artery disease,^3^ heart failure,^4^ or stroke,^5^ among others. The cited manuscripts are only a few examples of studies published between 2019 and 2020. Nevertheless, they are a representative set of the research conducted in the previous years and highlight the heterogeneity of methodologies used by different research groups. Indeed, previous studies have focused on serum, plasma, or extracellular vesicles (EVs). In addition to the biological bias introduced by the use of different matrices,^6^ there are also important technical biases linked to distinct isolation protocols.^7^ The differences between these protocols often lead to preferential isolation of EVs, platelets, and protein complexes that affects the relative concentrations of circulating miRNAs which ultimately limits the reproducibility.

Other preanalytical and analytical aspects must be considered,^8^ including the substantial technical variation associated with RNA isolation, the absence of robust internal controls, and the impact of factors such as age, sex, and pharmacological therapies on the miRNA levels.

## 2 POOR EXPERIMENTAL DESIGNS AND REDUCED SAMPLE SIZES

One of the most common experimental designs in the discovery starts from artificial case‐control approaches based on patients with different pathologies and healthy individuals to serve as controls. Although useful for molecular phenotyping, this approach implies an inadequate consideration of confounding factors, that is, treatments and comorbidities.

The low use of large and independent cohorts, together with the increased cost associated with the processing and analysis of a large number of samples, limits the translation of the experimental findings. The lack of replication of the results in independent cohorts also contributes to the skepticism toward the clinical applicability. Consequently, study power and sample size calculations should be a primordial early step in the study design.

## 3 ONGOING DEVELOPMENT OF TECHNOLOGY

A great challenge faced by the field is the lack of reproducibility across technologies for biomarker discovery. Although great advances have been made with the development of next‐generation sequencing platforms aimed to analyze ultra‐low starting quantities of RNA, most of these approaches are highly biased and hardly reproducible with other sequencing platforms, or even with more quantitative approaches such as PCR.^7^ The underlying reason behind this issue is dual: first, the low RNA input results in longer amplification cycles, where small biases are exponentially amplified; and second, the presence of overrepresented sequences with low‐to‐none clinical value often represents an important part of the sequencing endproduct.^9^

## 4 SIMPLE SIGNATURES AND NEED FOR ARTIFICIAL INTELLIGENCE INCORPORATION

A large percentage of previous studies have proposed the combination of a limited number of miRNAs to increase the discriminative potential of their biomarker set. Since diseases are consequence of abnormalities in the entire gene expression network, a more comprehensive strategy is fundamental. In this scenario, artificial intelligence tailored for biomarker discovery, including machine learning algorithms, has become a popular alternative statistical approach.

Artificial intelligence provides great advantages. The technique is not solely based on single‐circulating miRNAs, and/or sociodemographic, clinical, and pharmacological information, to discriminate cases from controls or predict disease risk or outcome, but it also weights and combines the value of several genes to produce a miRNA signature with predictive potential. This alternative has already yielded quite promising results in the evaluation of cardiovascular conditions.^10^

While these kinds of approaches are still in the early stages, the results produced in the upcoming years will certainly yield more robust biomarkers with stronger discriminative potential.

## 5 PERSPECTIVES

Despite the significant resources invested in miRNA‐focused research and the amount of available information, we are still far from their incorporation into routine clinical practice at the short‐ and medium‐term.

The clinical application of miRNAs in the long‐term requires additional efforts to overcome current weaknesses, should they be methodological, technical, or analytical. Harmonized miRNA isolation and quantification methods and the use of standard operating procedures are crucial to improve the reproducibility between independent investigations. As such, the establishment of guidelines on best practices is essential to augment the quality of the studies. Additionally, the development of automated and standardized assays, and the miniaturization of the methods, should be intensified for bench‐to‐bedside translation. The implementation in clinical laboratories also requires a detailed analysis of cost‐effectiveness. The support or creation of biobanks accessible to researchers should be promoted to address the low availability of large and independent cohorts.

To accomplish this, systematic and collaborative approaches, including basic researchers, clinical investigators, industry partners and governmental agencies, are fundamental to aid in translating biomarkers to the clinic. Overall, it is fundamental to create and improve the structures favoring the collaboration between the different stakeholders, in the addition to enhance the funding opportunities to facilitate the transition from bench to bedside.

## CONFLICT OF INTEREST

DdGC and YD hold patents related to diagnostic and therapeutic applications of RNAs.
